# Problems and Barriers Related to the Use of Digital Health Applications: Scoping Review

**DOI:** 10.2196/43808

**Published:** 2023-05-12

**Authors:** Godwin Denk Giebel, Christian Speckemeier, Carina Abels, Felix Plescher, Kirstin Börchers, Jürgen Wasem, Nikola Blase, Silke Neusser

**Affiliations:** 1 Institute for Healthcare Management and Research University of Duisburg-Essen Essen Germany; 2 QM BÖRCHERS CONSULTING+ Herne Germany

**Keywords:** mobile health, mHealth, app, Digital Health Application, DHA, Digitale Gesundheitsanwendungen, DiGA, problem, barrier, mobile phone

## Abstract

**Background:**

The digitization of health care led to a steady increase in the adoption and use of mobile health (mHealth) apps. Germany is the first country in the world to cover the costs of mHealth apps through statutory health insurance. Although the benefits of mHealth apps are discussed in detail, aspects of problems and barriers are rarely studied.

**Objective:**

This scoping review aimed to map and categorize the evidence on problems and barriers related to the use of mHealth apps.

**Methods:**

Systematic searches were conducted in the MEDLINE, Embase, and PsycINFO databases. Additional searches were conducted on JMIR Publications and on websites of relevant international organizations. The inclusion criteria were publications dealing with apps similar to those approved in the German health care system, publications addressing problems and barriers related to the use of mHealth apps, and articles published between January 1, 2015, and June 8, 2021. Study selection was performed by 2 reviewers. The manuscript was drafted according to the PRISMA-ScR (Preferred Reporting Items for Systematic Reviews and Meta-Analyses extension for Scoping Reviews) checklist. The analysis of the included publications and categorization of problems and hurdles were performed using MAXQDA (VERBI Software GmbH).

**Results:**

The database search identified 1479 publications. Of the 1479 publications, 21 (1.42%) met the inclusion criteria. A further 8 publications were included from citation searching and searching in JMIR Publications. The identified publications were analyzed for problems and barriers. Problems and barriers were classified into 10 categories (“validity,” “usability,” “technology,” “use and adherence,” “data privacy and security,” “patient-physician relationship,” “knowledge and skills,” “individuality,” “implementation,” and “costs”). The most frequently mentioned categories were use and adherence (eg, incorporating the app into daily life or dropouts from use; n=22) and usability (eg, ease of use and design; n=19).

**Conclusions:**

The search identified various problems and barriers in the context of mHealth apps. Although problems at the app level (such as usability) are studied frequently, problems at the system level are addressed rather vaguely. To ensure optimal use of and care with mHealth apps, it is essential to consider all types of problems and barriers. Therefore, researchers and policy makers should have a special focus on this issue to identify the needs for quality assurance.

**International Registered Report Identifier (IRRID):**

RR2-10.2196/32702

## Introduction

### Background

Since the development of the iPhone in 2007, the proliferation of apps has steadily increased. In particular, mobile health (mHealth) solutions, such as web applications or native apps, are increasingly diffused and provide many approaches to support users’ health. They can be applied in health monitoring and surveillance, for health promotion and raising awareness, communication and reporting, data collection, telemedicine, emergency medical care, point-of-care support, and decision support [1].

With the aim of benefiting from the potential of new technologies, such as mHealth apps for health care, the Digital Healthcare Act was introduced in Germany in December 2019. Hereby, particular mHealth apps with a low-risk class (I or IIa according to the Medical Device Regulation or, within the scope of the transitional provisions, the Medical Device Directive), known as *Digital Health Applications* (Digitale Gesundheitsanwendungen [DiGA]), became part of the German health care system [2,3]. During the corresponding approval process, the “Fast-Track Process for DiGA,” mHealth apps have to fulfill a predefined set of criteria. Among other things, these aim to prevent safety issues and problems with data privacy and security and to guarantee benefits either in the form of medical benefits or patient-relevant structure and process improvements for the patient [4]. mHealth apps that meet these requirements can be included in the DiGA directory. The apps listed in this directory are reimbursable by statutory health insurers. Currently, approximately 40 DiGA are listed and subsequently reimbursable. Although the German Fast-Track Process for DiGA is currently unique in the world, it has been announced that it will also be applied in France [5].

Many publications are addressing the possible benefits of mHealth apps. For example, mHealth apps for behavior change (either as a stand-alone intervention or as part of a larger intervention) have been shown to positively impact health outcomes compared with standard care and can be a useful adjunct in behavior change health interventions [6]. In addition, Liu et al [7] examined the effectiveness of mHealth apps for assisted self-care interventions in patients with type 2 diabetes, hypertension, or both and found that they were effective in improving blood glucose levels and blood pressure control. Wang et al [8] systematically reviewed the effectiveness of mHealth apps for monitoring and managing mental health symptoms or disorders and found that they have the potential to monitor or improve symptoms of certain mental health disorders, such as anxiety, stress, alcohol disorder, sleep disorder, depression, suicidal behaviors, and posttraumatic stress disorders. Finally, rising DiGA prescription numbers and strong interest from physicians and psychotherapists indicate that DiGA are expected to have the potential to improve care and, in some cases, fill existing gaps in care [9].

### Objectives

Nevertheless, as in other sectors and areas of health care, problems and barriers might arise in the context of mHealth apps. Therefore, an integrated application of mHealth in health care systems requires a comprehensive analysis of problems and barriers to adequately address potential challenges and risks in advance. To the best of our knowledge, problems and barriers related to mHealth have not yet been gathered systematically. Such a compilation would be the precondition to analyze whether certain problems require further governance and regulation during the processes of development, approval, dissemination, or use. Therefore, this study aimed to systematically search the literature to identify problems and barriers related to the use of mHealth apps similar to DiGA. The identified problems and barriers were compiled and categorized.

## Methods

### Overview

A scoping review was conducted to identify the problems and barriers related to the use of mHealth apps. This research was guided by the 5 mandatory stages for scoping reviews proposed by Arksey and O’Malley [10], which were further developed by Levac et al [11]. The manuscript was drafted according to the PRISMA-ScR (Preferred Reporting Items for Systematic Reviews and Meta-Analyses extension for Scoping Reviews) checklist [12]. The corresponding research protocol was published in *JMIR Research Protocols* [13].

### Search Strategy

A systematic search for articles published between January 1, 2015, and June 8, 2021, was conducted using bibliographic databases (MEDLINE, Embase, and PsycINFO). The search strategies were developed through discussion (GDG and CS) and with the aid of an experienced researcher (SN).

To develop a suitable search string for the systematic search in the MEDLINE, Embase, and PsycINFO databases, the methodology, issues, participants (MIP) scheme including methodology (all methodologies), issues (problems and barriers related to mHealth apps), and participants (main focus on patients and health care providers) was adapted [14]. Subsequently, the search terms and links between them were defined. Searches for defined terms were restricted to the occurrence in the abstract, title, and keywords. If there were indexing terms (Medical Subject Headings and Emtree), the search string was extended accordingly. The final search strategy for each database can be found in the research protocol [13].

Results were loaded into the EndNote reference management program (version X9; Clarivate). To supplement additional evidence, JMIR Publications was searched on January 18 and 19, 2022, and the reference lists of included studies were investigated on eligible articles.

The search in JMIR Publications was performed using the search function on the journal’s website. For this purpose, the problem terms were combined with either the term “mHealth app” or the term “mobile app.” This adjustment was made owing to a consensus paper recommended by the editor [15].

Apart from the bibliographic databases and reference lists, gray literature sources such as reports, guidelines, and working papers were searched via institutional websites. A full list of the institutions considered can be found in the corresponding research protocol [13].

The search for gray literature was conducted based on the institutional website in question. If available, search fields were used to identify publications using search words related to mHealth apps. Otherwise, relevant subpages with reference to the topic of mHealth apps were searched.

### Eligibility Criteria

The inclusion criteria were articles focused on problems and barriers related to the use of mHealth apps that were similar to the German DiGA concept. Journal papers were included if they were peer reviewed; published in 2015 or later; and were written in English, German, or French. Papers were included irrespective of their research method. See Textbox 1 or the research protocol [13] for the detailed inclusion criteria. The criteria that had to be met for mHealth apps to be classified as being similar to DiGA can be derived from the exclusion criteria in Textbox 2. Reviews and app assessments of mHealth app categories, which in principle could also be implemented as DiGA or have already been implemented, were also assessed as being similar to DiGA and consequently included as well.

**Inclusion criteria.**

- Articles mentioning problems and barriers related to the use of mobile health (mHealth) apps.
- A problem term mentioned in the abstract or title is related to the use of mHealth apps.
- Publication with a focus on mHealth apps.
- The included mHealth apps were similar to *Digitale Gesundheitsanwendungen*.
- Articles published in 2015 or later.
- Language: English, German, or French.

**Exclusion criteria.**

- Did not provide an answer to the research question.
- The problem term mentioned in the abstract or title was not related to the investigated mobile health (mHealth) app.
- Publication does not focus on mHealth apps.
- Examined mHealth apps fulfill ≥1 of the following criteria:
  Not used by the patient
  No relation to illness, injury, or disability
  Primary prevention
  The medical purpose is not achieved through the main digital functions
- Research protocol or conference abstract.
- Article published before 2015.
- Language other than English, German, or French.

Exclusion criteria were not providing an answer to the research question or not having at least one of the predefined problem terms related to the investigated app (“difficulty,” “obstacle,” “problem,” “issue,” “challenge,” or “barrier”) in the title or abstract. Further articles were excluded if the investigated mHealth apps were not similar to DiGA (not for patient use; no relation to illness, injury, or disability; for primary prevention; or not achieving its medical purpose through the main digital functions); the publication date was before 2015; or the language was other than English, German, or French. Furthermore, research protocols and conference abstracts were excluded from this study. The exclusion criteria are presented in Textbox 2 or in more detail in the research protocol [13].

### Article Screening and Data Extraction

Duplicates were removed after downloading citations and transferring them into EndNote. Screening and selection were performed in 2 steps. In a first step, 2 reviewers (GDG and CS) independently assessed the titles and abstracts. In a second step, articles included for full-text screening were independently assessed by the same reviewers using exclusion criteria (Textbox 2). In case of disagreement, conflicts were resolved by a third person (SN).

The 2 reviewers (GDG and CS) used MAXQDA (VERBI Software GmbH) to independently mark and extract relevant text characteristics of the included articles. A previously developed data-charting form was used for the extraction. Extracted data consisted of metadata, such as article characteristics as well as information related to the underlying research question—problems and barriers related to the use of mHealth apps. Thus, the relevant items were author, year, study country, study participants, type of study, underlying diseases, and problems and barriers related to the use of mHealth apps.

### Synthesis of Results

After evaluating the included studies, the results were summarized in a descriptive manner. The identified problems and barriers were grouped into clusters. Whenever a problem or barrier arose that could not be sorted into an existing cluster, a new cluster was created. Finally, the respective clusters were appropriately named according to the problems and barriers they contained. In addition, the results were summarized, systemized, and presented in Multimedia Appendix 1 [16-44] and Multimedia Appendix 2 [16-44].

## Results

### Selection of Sources of Evidence

**Figure 1:**
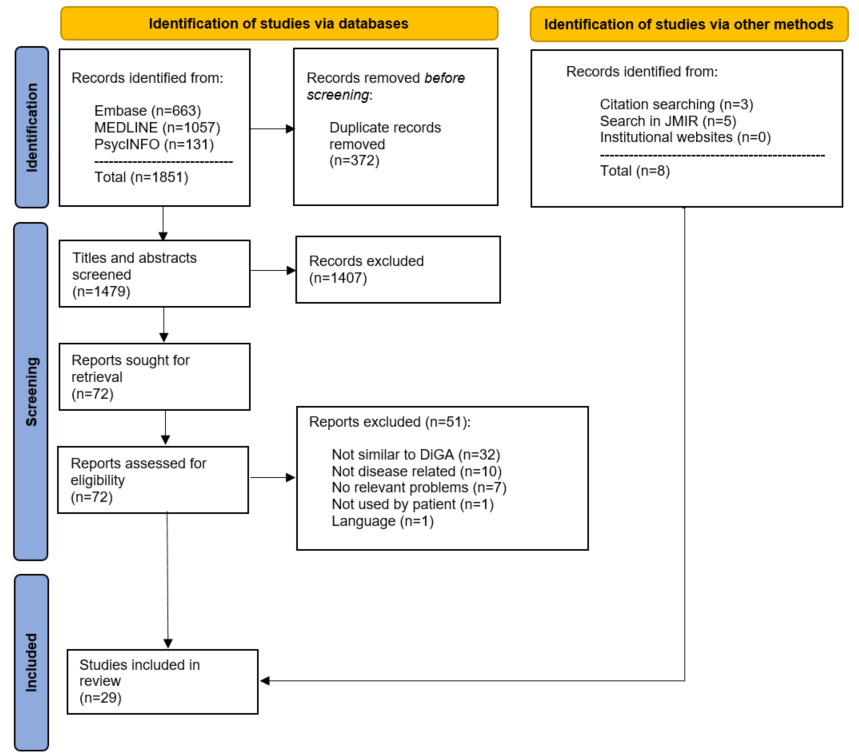
Flow diagram depicting the selection of sources of evidence. DiGA: Digitale Gesundheitsanwendungen.

The systematic search yielded 1479 articles after removing duplicates (Figure 1). Of these 1479 articles, 72 (4.87%) studies were screened in full text, and subsequently, 21 (1.42%) studies met the inclusion criteria [16-36]. Additionnaly, 3 studies were identified by screening the references of the included studies [37-39] and 5 studies were identified from the search in JMIR Publications [40-44]. The search on institutional websites did not yield any further results. Of the 1479 articles, 29 (1.96%) studies were included in this scoping review. The full-text screening process and reasons for exclusion are summarized in Multimedia Appendix 3 [16-36,45-95].

### Included Studies

Of the 29 articles included, 25 (86%) were primary studies [16,18-22,24-27,30-44] and 4 (14%) were nonsystematic reviews [17,23,28,29]. The United States (8/29, 26%) and the United Kingdom (5/29, 17%) were the countries with most included studies. All studies are listed in Multimedia Appendix 1. The included studies varied widely in terms of study design, underlying diseases, sample sizes, and sample composition. Furthermore, the objectives of the investigation varied. Of the 29 articles included, 18 (62%) articles focused on 1 app [16,18,19,22,25,26,30,32,33,36-44], 2 (7%) articles focused on multiple apps [24,35], and 9 (31%) articles did not focus on a specific app or device [17,21,23,24,27-29,34,35]; 8 (28%) studies included had a qualitative design [16,18,21,32,34,37,39,42], 5 (17%) studies were quantitative [22,24,27,31,35], 12 (41%) had a mixed methods design (qualitative and quantitative) [19,20,25,26,30,33,36,38,40,41,43,44], and 4 (14%) studies were reviews [17,23,28,29] (Multimedia Appendix 1). Most qualitative studies were based on interviews [16,26,30,32-34,39-42,44], focus group studies [18,21,36], or included both [19,38,43]. Quantitative research methods were mostly questionnaire studies [19,20,22,26,30,31,33,35,36,38,40,41,44]. Of the 29 articles included, 3 (10%) studies included a randomized controlled trial [19,20,38]. Overall, the studies included between 1 [37] and 1040 participants [31]. For further information on the included articles, refer to Multimedia Appendix 1.

### Synthesis of Results

#### Overview

**Figure 2:**
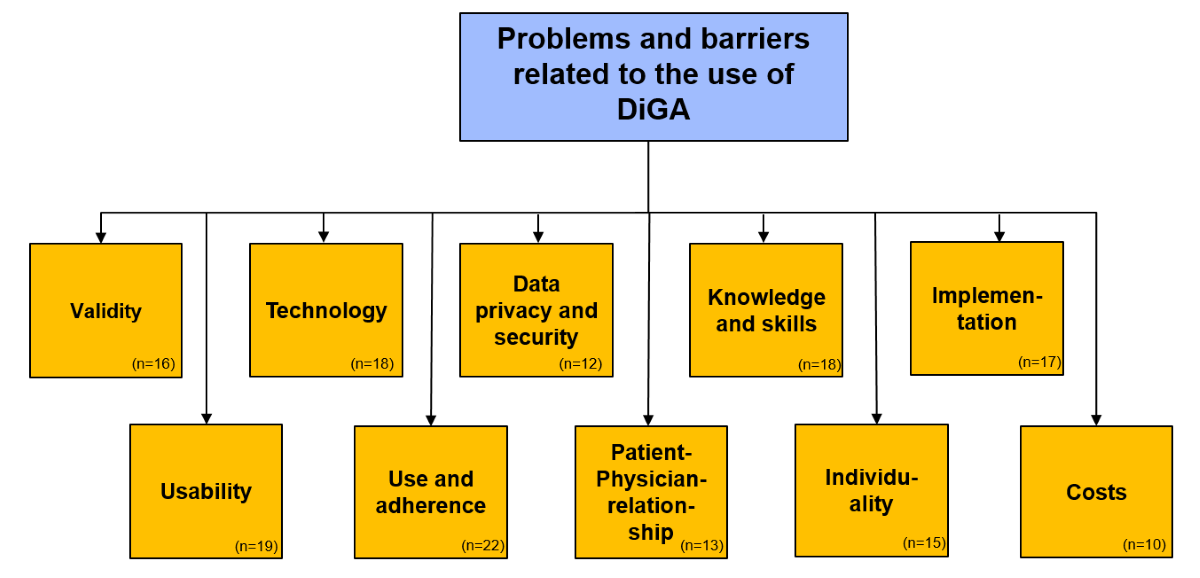
Problems and barriers related to the use of DiGA (n=number of included articles mentioning the respective category). DiGA: Digitale Gesundheitsanwendungen.

The problems and barriers identified in the included studies were categorized into 10 major groups. The included studies usually addressed several different problems and barriers. Multimedia Appendix 2 provides an overview of the categories of problems and barriers that were found in each article. The 10 groups included “validity,” “usability,” “technology,” “use and adherence,” “data privacy and data security,” “patient-physician relationship,” “knowledge and skills,” “individuality,” “implementation,” and “costs” (Figure 2).

#### Validity

Problems with validity were addressed in 16 articles [16,18,21,23-30,33,35,39,40,44]. Of these, 3 described quantitative studies [24,27,35], 4 described qualitative studies [16,18,21,39], 6 were mixed-design studies [25,26,30,33,40,44], and 3 were reviews [23,28,29]. Problems addressed in these studies were mainly in the area of contents, outcomes, and user input.

Problems in validity concerning the contents and outcomes of some mHealth apps were due to a lack of accordance with clinical standards. A fundamental problem was found in missing empirical evidence [24,27,29].

Some content was declared as inappropriate [18], wrong [24], or ambiguous [24,33,40]. Patients and health care staff stated that the depth and quality of information was often not suitable [21,39]. One quantitative study found that a few apps provided details about the underlying formulas used for calculations [24]. In 2 studies, some users criticized that functions did not meet their needs [35,40].

The lack of added value was perceived or assumed in qualitative and quantitative studies [21,25-27,44]. Some studies described mHealth solutions as inferior to usual care [21,23,29,30] or mentioned adverse effects or even harm [16,21,27,28,39]. One mixed-design study found that physiotherapists were skeptical about whether a hybrid setting including an mHealth app could be conducive to building and maintaining a robust working alliance between patients and physiotherapists [25].

In addition to problems with app content, an app assessment study found problems with user inputs and their validation [24]. In another study, patients described problems with changing the entered values [39]. Only a few apps provided guidance based on user-entered data. This was especially important in mental health crises or the risk of suicide [27]. Some apps caused inappropriate alerts after incorrect data entry in the settings component [44]. Patients manipulated the generated results by deliberately entering incorrect values to receive better feedback from the app [39]. Therefore, the medical benefits of the app could be reduced. Apps including physical exercises also faced the problem of validation. Lack of feedback on the correctness of exercise executions led to a feeling of insecurity and incorrect execution of exercises [25].

Health care professionals described app measurements, calculations, and the resulting data provided to the user as imprecise and inaccurate [16]. App assessment and interviews with patients revealed that incorrect results, despite correct input values, were a problem [24,39]. In 1 study, health care providers criticized that mHealth apps were not able to consider each aspect related to the subjects separately [33].

#### Usability

In total, 19 articles described the problems and barriers related to the usability of mHealth apps [16,18-20,22,25,26,29,30,33,35,37-44]. Two quantitative studies [22,35], 5 qualitative studies [16,18,37,39,42], 11 mixed-design studies [19,20,25,26,30,33,38,40,41,43,44], and 1 semistructured review [29] addressed usability as a problem. On the one hand, usability issues related to appearance or contents were seen in the software (the apps themselves), and on the other hand, problems were found in the hardware (executing devices). Some articles cited usability as an unspecified problem, among others [16,20,44].

Both patients and health care professionals described the design of mHealth apps as problematic [16,18,25,26,33,37,39,43,44]. Some apps included nonintuitive navigation or interfaces [26,29,37,38,44] or were generally difficult to use [18,19,22,30,35,41,42]. Three studies found that an insufficient or no user manual was provided [30,39,43]. Further issues with usability were observed in bulkiness or size of devices [19,37,40], visual unattractiveness, and discomfort [20].

#### Technology

Technical problems were mentioned in 18 articles [16,18-20,22,24-27,29,33,37-40,42-44] and were found in both devices and software. Three quantitative studies [22,24,27], 5 qualitative studies [16,18,37,39,42], 9 mixed-design studies [19,20,25,26,33,38,40,43,44], and 1 review [29] described problems related to technology.

A case study with an older user found a dependence on technological support [37]. Patients faced issues related to hardware, such as lack of free storage space on the smartphone, short battery life [20], and use of small devices with small screens, such as smartphones [44]. Some patients still use feature phones with limited functions compared with smartphones and accordingly cannot use apps [22].

Software-related issues were seen in functionality [27,43], challenges with software updates, and technical issues with operating systems [44]. Patients reported bugs, glitches, or intermittent screen freezes in 2 apps [16,44]. Technological failures may lead to physician-induced errors [33]. In addition to technological problems, it was stated that detecting these potential issues before app distribution is a challenge [24].

A further issue was observed in terms of compatibility. Problems for patients could result from incompatibility or difficulties between apps and running devices [18,20,26,38], between running and external devices [19,20], and connection to a server [40] or electronic health records [42].

Some articles mentioned technical problems that were not described further [19,25,29,37,39,44]. One mixed-design study referred to technical difficulties with access but did not provide further details [38].

#### Use and Adherence

Problems in the category of use and adherence were found in 4 quantitative studies [22,24,31,35], 6 qualitative studies [16,18,21,37,39,42], 9 mixed-design studies [19,20,25,26,30,38,40,43,44], and 3 reviews [17,28,29]. Qualitative research with patients as well as health care professionals and 1 review found a common problem of lack of adherence [16,29,39,42]. Many studies have reported high dropout rates [18-20,22,26,28-31,35,38,40]. In 2 studies, patients expressed minimal or no interest in the use of mHealth apps [42,44]. Problems cited regarding use and adherence included lack of motivation, additional burden on patients, social or disease context, lack of time and integration of the app into daily life, and lack of personalized elements.

Patients and health care professionals perceived lack of motivation [18,21] and lack of engagement in users [16,19,25,40,43] as reasons for low adherence and a high number of dropouts. In 1 study, users expressed that they had forgotten to use the app [20].

Some patients perceived the app as an additional burden or found it overwhelming [19,42,44]. Some participants did not update their goals to conform to expert recommendations and maintain achievable goals [19]. Otherwise, patients expressed concerns about being judged if they did not complete or missed lessons [37]. Two qualitative studies revealed that some patients did not use the app based on the given advice [39,42].

Some social situations [19,20,24,25,37] or disease-specific contexts [37] were reported as being problematic. Environmental influences [19,26] and special use cases [19] were further issues. One review stated that distraction by other web-based activities could be a problem [29].

Patients pointed out that the lack of a pause option [26] and difficulties with integration in everyday life can be a major barrier to adoption [44]. A further risk was a possible interference of technology use on relationships [40].

Lack of time was a major factor that was decreasing use [20,31,42,44]. Repetitive, long, complicated, and boring content reinforced the problem and might lead to even less time spent on mHealth apps [19,21,25,26,29,38].

Qualitative studies and reviews have shown that the lack of human factor also affected use and adherence. In other words, mHealth apps lacked personal touch, empathy, and complex aspects of human interaction [17,21]. Communication was sometimes seen as ineffective [29]. Some people will not use mHealth apps and reject them because they see their recovery as a process that depends only on the health care professionals caring for them [16].

#### Data Privacy and Security 

Data security and data privacy were addressed in 12 publications [16,18,21,27-29,33-35,40-42]. Thereof, 2 studies had a quantitative design [27,35], 5 studies had a qualitative design [16,18,21,34,42], 3 studies were mixed-design studies [33,40,41], and 2 publications included reviews [28,29]. There are mainly 2 groups of problems related to generated or collected data.

On the one hand, there are data security and data privacy problems [16,18,21,27,29,33-35,41,42]. On the other hand, there are problems with nontransparent communication of data privacy policies [21,27,28,40].

First, concerns regarding weak security arise in the context of data security and privacy [34,35]. Thus, a study of patients who are chronically ill found that 37.2% of them reported being concerned about the disclosure of personal information [35]. Further problems regarding data security and privacy were access without permission [16] and possible breaches of and concerns about confidentiality [16,27,33,39,42]. One app assessment study emphasized in its discussion that when data are stored on provider servers, there is an increased risk that the data will be used or sold for undesirable purposes [27]. Finally, health care practitioners mentioned problems regarding the identification of individuals by unauthorized data access [21] and patients worried about the unpredictable consequences of data leaks [34].

The other problem was the transparency of data handling [40]. Although some apps did not provide privacy policies [27,28], others were not clear or difficult to understand [21,27].

#### Patient-Physician Relationship

In this context, the patient-physician relationship refers to all types of relationships between patients and health care providers. Thus, it includes therapeutic relationships as well. Problems in this category were described in 13 articles [16,21,23,25,27,29-32,34,38,40,44] and included “the attempt to replace the clinician,” “lack of a therapeutic alliance,” “negative impact on the relationship,” “information inequalities,” and “the question of responsibility.” This category was addressed in 2 quantitative studies [27,31], 4 qualitative studies [16,21,32,34], 5 mixed-design studies [25,30,38,40,44], and 2 reviews [23,29].

Both patients and health care professionals mentioned problems with the lack of face-to-face contact. Both assumed preferences for face-to-face communication for some patients and providers [21,34]. Health care professionals have particularly emphasized the lack of nonverbal communication and para-communication associated with face-to-face conversations [21]. Nevertheless, the spectrum of physician replacement ranges from taking over individual decisions [40] to complete replacement [21,23,44]. One review described substantially lower treatment effects due to the substitution of face-to-face intervention [29].

The absence of a treating person resulted in a lack of therapeutic space [38] and therapeutic alliance [21,25] considered vital for successful therapeutic care [21]. Without human support, 1 study found difficulties with user engagement in active components [31].

Problems with the “patient-physician relationship” also occurred when mHealth apps were integrated into the treatment process. Even if technology can assist in health care, concerns regarding interference with relationships are pronounced by patients [34] and health care providers [21]. Physiotherapists saw that mutual trust could suffer from continuously monitoring a patient [25].

The limited capacity to export or download data reports reduces the ability to communicate directly from the app with others [27]. Information asymmetries can arise, and specialists could end up in situations in which patients receive treatment results before they do [32].

Finally, the responsibilities are altered and might lead to new problems. Physicians expressed concern that they have to handle additional data or alerts and that the use of mHealth apps could lead to the detraction of the patient’s self-management [16]. Physicians who did not engage as leaders in digital interventions were also seen as problematic in 1 study [30].

#### Knowledge and Skills

Problems and barriers related to knowledge and skills were identified in 4 quantitative studies [22,24,27,35], 7 qualitative studies [16,18,21,34,37,39,42], 4 mixed-design studies [19,30,33,41], and 3 reviews [17,23,29]. In some studies, it was found that patients had limited digital literacy [41] or abilities and experience regarding the use of mHealth apps [16,18,19,22,27,33,37]. In other cases, wrong perception [21,22,34,35] and a lack of knowledge [19,21,24,33,41] were seen as problematic.

Little, bad, or no experience with apps is seen as a major problem [18,19,42] and fosters the issue of low abilities and confidence with technology use [16,19]. Although young individuals showed few difficulties in app use, older patients, especially those with conditions such as dementia [27] or declining cognitive functions [37], face difficulties in app use [19]. A special problem is that older adult users have more problems because they use their mobile phones for known functions and discourage themselves from learning new technology through trial and error [22].

A major barrier for app use is found in its perception. Irrespective of individual apps, some patients believe that mobile phones [34] and apps [22,35] are complicated and difficult to use. Patients might also feel dismissed because they see inferior care in digital products compared with face-to-face contact [21].

In addition to the abovementioned problems in digital literacy [41], literacy in general and numeracy were found to be a barrier for app use [21,24]. In 2 studies, participants did not understand specific app functions [19,33].

Clinicians experienced similar problems as patients. Low experience and skills were frequently observed [16,19,21,23,29,30,39]. Some clinicians even had a more negative attitude toward this type of intervention than patients [17]. Others expressed a lack of confidence in the integration of technology in health care [21].

#### Individuality

A further problem mentioned in 15 articles [16,17,19,21,25,26,29,33,35-37,39,40,43,44] is the intention or capability to customize mHealth apps to the individual needs of patients. One quantitative study [35], 4 qualitative studies [16,21,37,39], 8 mixed-design studies [19,25,26,33,36,40,43,44], and 2 reviews [17,29] included problems and barriers corresponding to individuality. This is, for example, expressed in the fact that mHealth apps are usually not adapted to each individual [17,19,33,35-37,40,44]. Thus, the authors discussed the difficulty in designing attractive and useful programs for all patients, which are at least as effective as standard therapy [17,36], and pronounced the difficulties due to the diversity of the target users, especially in terms of age [33,35,37] and diseases [35]. Furthermore, the authors described individualization of functions due to perceptual impairments [37] and motor or physical issues [44] to be problematic. In 1 study, patients indicated that the goals set by the app were too simple and that the app could not be customized to their needs as much as necessary [40].

Different functions are affected by a lack of individualization. Patients and health care professionals expressed that exercise programs often consist of a fixed number of different standard exercises [16,25,26], that data input is limited to imprecise standardized possibilities [39], and that communication provided by the mHealth app is unadjusted [21,43,44]. A special problem is the “cold start problem.” It describes the need for time at the beginning of the intervention to personalize the app content to the user profile through artificial intelligence [26].

#### Implementation

The implementation of mHealth apps in health care systems faces different problems. Problems related to implementation were found in 4 quantitative studies [22,24,31,35], 4 qualitative studies [16,21,39,42], 5 mixed-design studies [30,38,40,41,44], and 4 reviews [17,23,28,29].

Barriers to access were seen as a problem for implementation. These occurred because of a lack of infrastructure, socioeconomic conditions, or social reasons. Lack of access (eg, lack of smartphones or broadband and computers) is a fundamental barrier for the use of mHealth apps [22,35,38,42]. Disparities in access subsequently foster concerns that only a fraction of users benefit from apps in health care [21,22,29]. Issues in the context of equity may stem from income or disability and result in nonequally distributed devices and connectivity [28]. Further barriers to access concerned stigma and culture [31] as well as language [28,31]. However, no further information was given on these barriers.

Further problems concerned transferability of study effects to real-world care and organizational barriers, such as lack of capacity or preparedness of health care systems and reimbursement structures. Successful transfer into clinical practice was seen as a problem [17,23,41]. Many questions, for example, regarding modes of action or for which target groups app-based therapy is most suitable, are still unanswered. Thus, mHealth programs showing effectiveness in experimental settings do not necessarily show good results in real health care settings [17]. Staff members reported low expectations and low confidence in the ability of national health care systems to implement digital tools [21]. Barriers for implementation were lack of health system readiness, organizational resistance to change, and policy uncertainties [44]. Approval of apps, for example, by the US Food and Drug Administration, focuses on safety and minimal effectiveness thresholds and does not provide sufficient information for decision makers [28]. Reimbursement options are not uniform [28], and a lack of collaboration among stakeholders, such as developers, health care professionals, and patients, in the design and development process affects acceptance and adoption [16].

Low acceptance is a 2-fold problem. On the one hand, some professionals have less interest in information in apps than in paper-based information. This was highlighted by patients commenting that care providers always asked for paper forms despite information being provided in app format [39]. On the other hand, professionals need to be open to the use of mHealth apps because their strong leadership engagement and promotion are fundamental for mHealth use [30,35].

Health care professionals see the use of digital solutions as an additional burden placed on them [21,40,42] and express fear regarding the complexity of, and the responsibility for, identifying and managing risk [21]. Interacting with mHealth apps was frequently seen as obstructive for workflows [21,28,42].

Three further problems concerning the implementation were as follows: first, some app manufacturers were not available and did not respond to requests [24,42]; second, some users expressed the amount of choice being overwhelming [16]; and finally, frequent app updates, requiring evaluation of new and confirmation of old functions, were potential problems [24].

#### Costs

Costs were mentioned in 10 articles [18,21,27-29,31,34,35,42,44]. Of these, 3 had a quantitative design [27,31,35], 4 were qualitative studies [18,21,34,42], 1 had a mixed-design [44], and 2 were reviews [28,29]. On the patient’s side, the use of mHealth apps might be problematic because it always requires a running device [21] and often requires data transmission [34,35]. One study mentioned potential costs for apps as a concern [18]. Furthermore, a problem for patients is the lack of opportunity to test and evaluate apps before they are purchased [27]. These costs usually have to be borne by patients and might lead to socioeconomic inequalities [21]. Another problem for patients is the lack of opportunity to test and evaluate apps before purchasing them [27].

As with traditional health services, health care practitioners need time to integrate mHealth apps into their treatment. However, this effort is often not reimbursed [28,29,42,44]. Therefore, providers demanded that the time used for mHealth interventions be compensated in the same way as face-to-face treatments [28]. Some clinicians questioned the value of investing in mHealth apps and preferred investing in staff training and staff employment rather than digital tools [21]. One article did not specify the problem of costs [31].

## Discussion

### Principal Findings

This scoping review maps the evidence on potential problems and barriers related to the use of mHealth apps fulfilling the basic criteria of DiGA. The inclusion criteria were as follows: (1) low-risk class (I or IIa); (2) use by the patient; (3) relation to illness, injury, or disability; (4) not for primary prevention; and (5) the medical purpose is achieved through the main digital function. To the best of our knowledge, this is the first scoping review on this topic.

In total, 29 studies on mHealth apps met the inclusion criteria. The included studies showed large heterogeneity, and identified problems and barriers were often only a by-product in the included articles.

Most of the studies originate from English-speaking countries. Thereof, 8 originated from the United States [18,27-31,42,43], 5 from the United Kingdom [19,21,24,40,41], and 2 from Ireland [24,28]. Four studies were conducted in Asian countries (Multimedia Appendix 1). Two of these were from China [35,37] and Korea [22,34]. Despite the presupposed DiGA similarity of the apps described in the studies, none of the included studies were from Germany.

The included studies differed substantially in terms of study design (Multimedia Appendix 1). A total of 8 studies had a qualitative design, and 5 studies had a quantitative design. Furthermore, 12 mixed methods studies and 4 reviews were included. Most of the included studies used interviews [16,19,25,30,32-34,38-44], questionnaires [20,22,30,31,33,35,36,38,40,41], and focus groups [18,21,36,38,43]. Only 3 studies used a randomized controlled trial [19,20,38].

The study populations investigated also varied widely (Multimedia Appendix 1). Although some studies focused on relatively balanced study populations [31], other studies included very specific populations, such as people with a military background with posttraumatic stress disorder [30] or an older woman with insomnia [37].

Identification of relevant aspects and categorization of problems and barriers was independently done by 2 reviewers. The categorization was performed by clustering aspects into consistent groups. New groups of problems and barriers were compiled if an identified problem could not be matched with the existing groups. This proceeding revealed 10 major categories of problems and barriers on a super ordinated level: “validity,” “usability,” “technology,” “use and adherence,” “data privacy and data security,” “patient-physician relationship,” “knowledge and skills,” “individuality,” “implementation,” and “costs.”

The categorization into the 10 problem groups is an approach to systematize problems and barriers in the context of mHealth use identified in the literature. In addition to the categories defined by the scoping review, it would be possible to include further categories or subcategories. For example, “demographics” could be such a category. This could include identified problems, such as the problems of older patients using the app or socioeconomic inequalities that pose problems to access. Although it would, in principle, be conceivable to define other problem categories, our research approach has proven to be well suited to identify relevant categories. All the identified problems and barriers could be clearly assigned to a category.

In addition, “actuality” would be another problem category that could be considered, as outdated content or technology could, in the worst case, lead to a compromise of patient safety. Unfortunately, in this review, no relevant texts, including this type of problem, were identified. Our final 10 categories were formed qualitatively based on the available evidence and should be used as a fundamental basis for further discussion and research.

While conducting the scoping review and interpreting the results, it was suggested that there might be some correlations between the problem categories. Thus, 1 category might have a direct influence on another category. For example, such correlations were suspected between “use and adherence” to mHealth apps and their “usability” or between “implementation” and “knowledge and skills.” However, as these are not confirmed results of the scoping review, these assumptions should be pursued in further studies.

Our results show that research in the area of problems and barriers is still rare compared with research on opportunities and possibilities. The problem categories identified can be attributed to the mHealth apps themselves, on the one hand, and their integration into the health care system, on the other hand. Regarding app-level problems (eg, “validity,” “usability,” “technology,” and “data privacy and data security”), there are already quality assessment tools especially developed for mHealth apps [96-99] that aim to ensure the quality of apps. Other issues such as “use and adherence,” “patient-physician relationship,” “knowledge and skills,” “implementation,” and “costs,” affect the entire health care system. In contrast to quality assurance approaches at the app level, such approaches do not yet exist at the system level. Further research is needed, particularly in this area. Only if the integration of mHealth apps into the health care system succeeds as a whole will patients sustainably benefit from the new technology. To achieve this goal, it is mandatory to explore those problems and barriers affecting various stakeholders. Not only scientists but also policy makers should have a special focus on these types of issues and address them within research and regulations.

In Germany, mHealth apps applying for the DiGA directory are initially examined for safety and suitability for use, data protection and information security, interoperability, robustness, consumer protection, ease of use, support of health care providers, quality of medical content, and patient safety, as well as evidence of positive health care effects [4]. Other categories of problems, such as “use and adherence,” “patient-physician relationship,” and especially “implementation” and “costs” are not sufficiently addressed, especially in high-quality studies, and need further investigation.

The problem categories identified can serve as a starting point for further research. For the less well-studied ones, systematic studies of higher quality and scoping reviews should delineate the field; for the better-studied problem categories, such as “validity,” “usability,” “technology,” and “data privacy and data security,” systematic reviews might be more useful to gain insights. However, in addition to further reviews in this area of research, it is important to consider the results of primary studies.

### Limitations

This review had some limitations. The search was not restricted to certain study types to capture a broad evidence base and include aspects currently under discussion. Therefore, in addition to quantitative and qualitative studies, narrative reviews were included, as long as they met the inclusion criteria. Often, the included articles addressed the problems and barriers only incidentally. Only including these different types of items enabled us to create a broad evidence base. This might be a starting point for further research on certain categories such as implementation.

As the systematization of individual problems has taken a lot of time, more recent evidence should also be examined. However, here the approach of examining problem categories should be explicitly pursued.

During the screening process, we did not determine the agreement between the 2 reviewers or the κ coefficient. Nevertheless, in cases of disagreement, we involved a third person. Thus, the inclusion or exclusion of texts was done qualitatively. Furthermore, we have listed the studies that were excluded in the full-text screening with the reason for each in Multimedia Appendix 3 to make our investigation more transparent and comprehensible to third parties.

### Conclusions

The findings of this scoping review are relevant not only for DiGA but also for all kinds of mHealth apps. Ten categories of problems and barriers were identified. Issues at the app level such as “validity,” “usability,” “technology,” “data privacy and security,” and “individuality” are addressed in several studies and are partly considered in quality assurance systems; problems and barriers related to the level (“use and adherence,” “patient-physician relationship,” “knowledge and skills,” “implementation,” and “costs”) of health care system are rarely extensively studied. Further research is essential to optimize the integration of mHealth apps into health care, especially in the area of system-related problems. In addition to serving as a starting point for further research, it is imperative that identified problems and barriers are considered in the development of new mHealth apps.

## Appendix

Multimedia Appendix 1 Overview of the included articles.

Multimedia Appendix 2 Problems mentioned in the articles.

Multimedia Appendix 3 Studies assessed in the full-text screening and exclusion criteria.

## Abbreviations

DiGA: Digitale Gesundheitsanwendungen
mHealth: mobile health
MIP: methodology, issues, participants
PRISMA-ScR: Preferred Reporting Items for Systematic Reviews and Meta-Analyses extension for Scoping Reviews
