# Supplementary material for: Problems and Barriers Related to the Use of Digital Health Applications: Scoping Review
Source: J Med Internet Res. 2023 May 12;25:e43808. doi: 10.2196/43808 (PMC10221513; doi:10.2196/43808)
Supplement: Multimedia Appendix 1 [file jmir_v25i1e43808_app1.docx]

## Appendix 1: Overview on included articles

| No. | **Author(s)** | **Year** | **Country** | **Field of application** | **Object of investigation** | **Intervention** | **Study type / Study design** | **Study population** | **Problem and Barrier categories addressed** | **Comments** |
| --- | --- | --- | --- | --- | --- | --- | --- | --- | --- | --- |
| 1 | Argent et al. [16] | 2018 | Ireland | Orthopedic rehabilitation | **1 app + 1device**  (prototype wearable exercise biofeedback system) | Demonstration of a prototype wearable exercise biofeedback system and subsequent one-to-one interviews | **Qualitative:**  Semi-structured interviews | **Health care professionals:**  10 healthcare professionals involved in the orthopaedic rehabilitation pathway (f: 6):  4 psychotherapists, 2 specialist nurses, 2 orthopedic assistants, 1 occupational therapist,  1 ward nurse | - Validity - Usability - Technology - Use & Adherence - Data Security & Data Privacy - Doctor-patient relationship - Knowledge & Skills - Individuality - Implementation | - |
| 2 | Barceló-Soler et al. [17] | 2019 | Spain | Depression | **No specific app or device** | Does not apply! | **Review:**  Narrative review | Does not apply! | - Use & Adherence - Knowledge & Skills - Individuality - Implementation | Description of the current situation in Spain regarding the use of internet psychotherapy programs for the treatment of depression in primary care |
| 3 | Beer et al. [18] | 2020 | USA | Lung cancer | **1 app**  (prototype mHealth app) | A video of the app prototype was shown to the participants. Subsequent semi-structured discussion was facilitated by moderators. | **Qualitative:**  Semi-structured focus groups | **Patients:**  19 patients or family members (f: 11):  11 lung cancer survivors  8 family members | - Validity - Usability - Technology - Use & Adherence - Data Security & Data Privacy - Knowledge & Skills - Costs | - |
| 4 | Bentley et al. [19] | 2020 | UK | COPD | **1 app +**  **1 device**  (smartphone app + activity tracker) | Intervention: Used the app and activity tracker  Control: Wore a blinded activity tracker  The technology was demonstrated to the participants and baseline activity goals were set within the app. The app was used throughout pulmonary rehabilitation and for 8 weeks afterward. | **Quantitative and Qualitative:**  Patients: Randomized controlled trial (RCT) and semi-structured interviews.  Questionnaire-based and physical activity-based outcome measures were taken at baseline, the end of pulmonary rehabilitation, and the end of maintenance.  Health care professionals: interviews and focus group discussion. | **Patients:**  30 patients (f: 17):  19 intervention group  11 control group  **Health care professionals:**  5 health care professionals | - Usability - Technology - Use & Adherence - Knowledge & Skills - Individuality | 47% of participants of the intervention group and 46% of the control group withdrew |
| 5 | Browne et al. [20] | 2020 | Ireland | Obesity | **2 apps +**  **1 device**  (2 smartphone apps (Mandolean and myBigO) + smart watch + food scale connected via Bluetooth with the mandolean app) | Intervention: Participants were guided through a practice setting using the first app with the food scale (Mandolean). Subsequently they were registered with the other app (myBigO).  Control: Usual care | **Quantitative and Qualitative:**  RCT, questionnaires and discussions  2 weeks of testing, including behavioural and quality of life questionnaires, anthropometry, rate of eating, and physical activity at baseline and after the 4-week treatment period.  Process evaluation was conducted to measure recruitment, study retention, fidelity parameters, acceptability, and user satisfaction. | **Patients:**  20 patients (9-16 years) with clinical obesity:  8 intervention group  12 control group | - Usability - Technology - Use & Adherence | 63% of participants of the intervention group and 25% of the control group withdrew |
| 6 | Bucci et al. [21] | 2019 | UK | Early psychosis | **No specific app or device** | Does not apply! | **Qualitative:**  Semi-structured focus groups | **Health care professionals:**  48 staff members from specialized mental health services (f: 27) | - Validity - Use & Adherence - Data Security & Data Privacy - Doctor-patient relationship - Knowledge & Skills - Individuality - Implementation - Costs | Opinions of frontline staff regarding the utility and appropriateness of using digital tools in the healthcare pathway for people accessing specialist secondary care mental health services. |
| 7 | Chung et al. [22] | 2020 | Korea | Insomnia | **1 app**  (Mobile phone-based cognitive behavioral therapy for insomnia app) | Participants were asked to use and test the app together with therapists and volunteer workers.  A part of the participants was enrolled in a 1-week home-based self-help cognitive behavioural therapy for insomnia with the app. | **Quantitative:**  Questionnaires  Questionnaires on sociodemographic characteristics, subjective evaluation of mental health status, and app usability after gaining hands-on experience (2-hour training program) with the app.  Questionnaires on subjective evaluation of sleep quality after the 1-week intervention. | **Patients:**  One-day training program with the app:  40 participants (>60 years) (f: 35)  One-week self-help intervention with the app:  9 female participants | - Usability - Technology - Use & Adherence - Knowledge & Skills - Implementation | - |
| 8 | Cuijpers et al. [23] | 2017 | Nether-lands | Depression | **No specific app or device** | Does not apply! | **Review:**  Narrative review | Does not apply! | - Validity - Doctor-patient relationship - Knowledge & Skills - Implementation | Synthesized knowledge on effectiveness and integration of internet-based therapies in the context of depression treatment |
| 9 | Huckvale et al. [24] | 2015 | UK | Diabetes | **No specific app or device^1^**  (46 apps were included) | Does not apply! | **Quantitative:**  App assessment:  Researchers identified and assessed apps on predefined criteria (eg. Included clinical disclaimer, documented calculation formula, lacked input validation) | Does not apply! | - Validity - Technology - Use & Adherence - Knowledge & Skills - Implementation | A systematic assessment of English-language rapid/short-acting insulin dose calculators available for iOS and Android was performed |
| 10 | Kowatsch et al. [25] | 2021 | Not specified – Authors from Switzer-land, Singapore and Korea | Diseases of the musculo-skeletal system | **1 app**  (mobile hybrid ubiquitous coaching app with additional augmented reality hardware) | Four loops including design and evaluation were conducted with an interdisciplinary team to assess perception by patients and physiotherapists. | **Qualitative and Quantitative:**  Observational study, semi-structured interviews and survey  Various surveys after each development loop. | **Patients:**  35 patients (study 1) + 15 patients (study 3)  **Health care professionals:**  11 physiotherapists (study 2) + 2 physiotherapists (study 3),  1 patient-physiotherapist dyad, 3 physiotherapists (study 4) | - Validity - Usability - Technology - Use & Adherence - Doctor-patient relationship - Individuality | - |
| 11 | Luna-Perejon et al. [26] | 2019 | Spain, Greece and Taiwan | Smoking cessation | **1 app**  (mobile Android app) | Group 1: The first group (experts) was informed about the steps required to properly use the app. Experts recruited further participants and passed on information to their recruited users.  Group 2: Smokers using Android smartphones recruited by experts | **Qualitative and Quantitative:**  Observational study, questionnaires, expert reports  Evaluation after the three-week testing period. App behaviour analysis was collected by the app. | **Patients:**  70 participants:  25 experts (f:11)  45 users | - Validity - Usability - Technology - Use & Adherence - Individuality | The "expert" group included smokers and non-smokers with enough knowledge to assess the app using an expert heuristic evaluation.  The second group were smokers. |
| 12 | Minen et al. [27] | 2021 | USA | Stroke, migraine, depression, alzheimer’s disease, dementia and anxiety | **No specific app or device^2^**  (83 apps for the top neuro-psychiatric conditions) | Does not apply! | **Quantitative:**  App assessment:  Researchers identified and assessed apps on predefined criteria (eg. Included functions, behaviour change rewards, and information about intervention, privacy policy, and payment) | Does not apply! | - Validity - Technology - Data Security & Data Privacy - Doctor-patient relationship - Knowledge & Skills - Costs | - |
| 13 | Mohr et al. [28] | 2021 | USA | Mental health | **No specific app or device^3^**  (Digital mental health treatments) | Does not apply! | **Review:**  Review within a forum | **Health care professionals**  Uncertain number of participants:  National and international expert stakeholders representing health care organizations, insurance companies and payers, employers, patients, researchers, policy makers, health economists, and digital mental health treatment companies | - Validity - Use & Adherence - Data Security & Data Privacy - Implementation - Costs | Forum meetings with expert stakeholders were hold to review the evidence of digital mental health treatments and to identify challenges to successful and sustainable implementation. |
| 14 | Okorodudu et al. [29] | 2015 | USA | Overweight, obesity | **No specific app or device^4^**  (web-based, mHealth, and exergames interventions) | Does not apply! | **Review:**  Semi-structured review | Does not apply! | - Validity - Usability - Technology - Use & Adherence - Data Security & Data Privacy - Doctor-patient relationship - Knowledge & Skills - Individuality - Implementation - Costs | A literature search was conducted to identify new approaches to promote behaviour changes in the management of obesity. |
| 15 | Possemato et al. [30] | 2017 | USA | Post-traumatic stress disorder | **1 app**  (self-management mobile app) | Development and refinement process of the intervention.  The intervention was delivered to veterans by clinicians. | **Quantitative and Qualitative:**  Feedback, questionnaire and interview  After 8 weeks of intervention veterans completed a satisfaction questionnaire and a patient feedback interview. | **Patients:**  Veteran feedback:  9 patients  **Health care professionals:**  Stakeholder Interviews:  9 providers  Clinician feedback:  3 clinicians | - Validity - Usability - Use & Adherence - Doctor-patient relationship - Knowledge & Skills - Implementation | The study was divided in three phases   1. Stakeholder interviews regarding implementation constructs 2. Patients provided feedback after receiving the intervention 3. Clinicians provided feedback after delivering the intervention |
| 16 | Pratap et al. [31] | 2018 | USA | Depression | **3 apps**  (different self-guided mobile apps) | Participants had to choose two of the three apps. Subsequently they were randomly assigned one of the two apps preferred and were asked to use it for four weeks | **Quantitative:**  Questionnaires and passive data collection  Mental health status and self-reported disability were assessed at baseline and subsequently at weekly intervals for 12 weeks. Daily changes in mood were assessed as well. Phone use was assessed by passive data collection. | **Patients:**  1040 participants (f: 266) | - Use & Adherence - Doctor-patient relationship - Implementation - Costs | Only 33.46% of the initially enrolled participants were active in the study.  Aim of this study was to compare recruitment and engagement in a fully remote trial (via custom apps). Secondary outcomes were treatment outcomes. |
| 17 | Ravn Jakobsen et al. [32] | 2018 | Denmark | Osteoporosis | **1 app**  (mHealth app to support women in self-management) | Does not apply! | **Qualitative:**  Field studies and semi-structured interviews. | **Patients:**  6 women with newly diagnosed osteoporosis,  **Health care professionals:**  2 scientists,  4 physicians,  2 laboratory specialists,  1 nurse,  1 nutritionist,  2 app developers | - Doctor-patient relationship | Three workshops were conducted with participants. |
| 18 | Sobrinho et al. [33] | 2018 | Brazil | Chronic kidney disease | **1 app**  (mHealth app to assist self-monitoring) | Patients used the app freely under observation. | **Quantitative and Qualitative:**  Interviews, questionnaire, observation  The questionnaire was used to guide the semi-structured interview. | **Patients:**  Usability test:  8 patients (f: 7)  **Health care professionals:**  Interviews:  1 nurse,  4 nephrologists  Evaluation:  Records of 60 patients by 3 nephrologists | - Validity - Usability - Technology - Data Security & Data Privacy - Knowledge & Skills - Individuality | Medical guidelines were reviewed and interviews conducted to determine requirements. Subsequently an mHealth app was developed and evaluated. |
| 19 | Son et al. [34] | 2020 | Korea | Chronic heart failure | **No specific app or device^5^**  (mHealth heart failure self-care interventions) | Does not apply! | **Qualitative:**  Semi-structured interview | **Patients:**  20 patients with diagnosis of chronic heart failure (f: 8) | - Data Security & Data Privacy - Doctor-patient relationship - Knowledge & Skills - Costs | Patients were interviewed about demand for reliable and customized health information, valuable features of mobile phone applications, barriers towards and expected benefits of using mobile health technology. |
| 20 | Sun et al. [35] | 2017 | China | Chronic diseases (cardiovas-cular disease, cerebrovas-cular disease, respiratory disease and diabetes mellitus) | **No specific app or device^6^**  (physical activity apps) | Does not apply! | **Quantitative:**  Questionnaires  The survey was conducted once. | **Patients:**  218 patients (f: 133) | - Validity - Usability - Use & Adherence - Data Security & Data Privacy - Knowledge & Skills - Individuality - Implementation - Costs | This study investigates on the current usage, willingness to use and barriers to using physical activity apps of Chinese patients with chronic diseases. |
| 21 | Wirken et al. [36] | 2018 | Nether-lands | Mental health of kidney donors and kidney donor candidates | **1 app**  (internet-based cognitive-behavioural therapy) | After an introduction living kidney donor population used the internet-based therapy. (Mean duration of 11.6 weeks) | **Quantitative and Qualitative:**  Semi-structured focus groups, questionnaires, technical data (eg. The frequency of logins)  Health related quality of life, anxiety and depression were measured before and after the internet-based cognitive-behavioural therapy. Other measurements were collected after finishing the intervention. | **Patients:**  Focus groups:  13 donors postdonation (f: 9)  Feasibility testing  8 eligible participants with higher risk profile  **Health care professionals:**  Focus groups:  1 nephrologist,  1 coordinating nurse,  3 medical social workers | - Individuality | Development and evaluation of a therapist-guided internet based cognitive-behavioural therapy intervention. |
| 22 | Chen et al. [37] | 2016 | China | Psycho-physiological insomnia | **1 app**  (mHealth app-assisted cognitive behavioral therapy) | The app was applied in a six-session treatment. | **Qualitative:**  Case study | **Patients:**  One 64-year-old-Chinese woman | - Usability - Technology - Use & Adherence - Knowledge & Skills - Individuality | The utilization, advantages, and limitations were investigated. |
| 23 | Hatcher et al. [38] | 2018 | New Zealand | Depression or dysthymia | **1 app**  (web-based therapy supported by a coach) | Intervention: usual care and the web-based therapy supported by a coach.  Control: Usual care and information about web resources. | **Quantitative and Qualitative:**  RCT, observation, questionnaire, structured interviews, focus groups  Primary outcome (self-rating scale for depression) was measured after 12 weeks.  Secondary outcomes (SF-36, EQ-5D, medication use, time to first outpatient appointment, and the number of outpatient appointments) were measured at baseline, after 2 weeks, 6 weeks, and 12 weeks. | **Patients:**  Randomized-controlled-trial:  63 participants (f: 34)  Interviews:  9 participants (f: 4)  2 focus groups (number of participants not specified) | - Usability - Technology - Use & Adherence - Doctor-patient relationship - Implementation | 37% of the intervention group and 21% of the control group dropped out during follow-up. |
| 24 | Skar et al. [39] | 2017 | Norway | Gestational diabetes mellitus | **1 app**  (mHealth app for disease management) | Does not apply! | **Qualitative:**  Semi-structured interviews | **Patients:**  17 patients | - Validity - Usability - Technology - Use & Adherence - Knowledge & Skills - Individuality - Implementation | Interviews were conducted to explore the experiences of participants with using the app to control their blood glucose values and to receive health and nutrition information. |
| 25 | Kellett et al. [40] | 2020 | UK | Anxiety and Depression, borderline personality disorder, and narcissistic personality disorder | **1 app**  (mHealth app for cognitive analytic therapy) | Patients used the mHealth app within a case series design. | **Quantitative and Qualitative:**  Semi-structured interviews, questionnaire, passive data collection via app  The questionnaire on clinical outcomes was completed at baseline and termination of the intervention. | **Patients:**  Case series design:  10 patients:  5 anxiety and depression,  1 borderline personality disorder,  1 narcissistic personality disorder  Interviews:  6 of the patients included  **Health care professionals**  Interviews:  3 therapists | - Validity - Usability - Technology - Use & Adherence - Data Security & Data Privacy - Doctor-patient relationship - Individuality - Implementation | 3 patients dropped out early  The relationship between app use and clinical outcome was investigated. Therefore, measurements were taken pre- and post-intervention. Additionally interviews were conducted with patients and therapists to explore the experiences of using the app. |
| 26 | Hardy et al. [41] | 2018 | UK | Nonaffective psychosis | **1 app**  (web app including blended digital therapy) | Patients used the app within a case series design. | **Quantitative and Qualitative:**  Questionnaires, semi-structured interviews  Assessments were conducted at baseline, post therapy (8weeks), and at follow-up (12 weeks). | **Patients:**  18 participants in the design research sample of which 12 completed the case series | - Usability - Data Security & Data Privacy - Knowledge & Skills - Implementation | A case series design including a brief protocol-based therapy with assessments at baseline, post therapy and at follow-up, was conducted. Therapy feedback was collected regarding acceptability, usefulness, and usability. In a second step design research was conducted to develop the app. |
| 27 | Thies et al. [42] | 2017 | USA | Diabetes and/or hypertension | **1 app**  (mHealth app) | Does not apply! | **Qualitative:**  Semis-structured interview | **Patients:**  8 patients  **Health care professionals:**  1 primary care provider,  2 nurses,  2 research assistants | - Usability - Technology - Use & Adherence - Data Security & Data Privacy - Knowledge & Skills - Implementation - Costs | The original study was suspended due to low enrollment and inconsistent use of the app by enrolled patients  This study was conducted to explore reasons for the unsuccessful trial. |
| 28 | Thirumalai et al. [43] | 2018 | USA | Multiple sclerosis | **1 app**  (mHealth app for telerehabili-tation) | Participants had to perform usability tests under observation and were subsequently asked to rate usability. | **Quantitative and Qualitative:**  Focus group, observation, Interview | **Patients:**  Focus group:  8 patients (f: 6)  Usability test 1:  8 patients (f: 4)  Usability test 2:  5 patients (f: 4) | - Usability - Technology - Use & Adherence - Individuality | The development process included two phases:   1. Ground-level creation of app features and exercise content (focus-group) 2. Proof of concept trials through individual user testing (usability testing) |
| 29 | Woods et al. [44] | 2019 | Australia | Heart failure | **1 app**  (mHealth app for self-management) | Patients used the app for 14 days in a home setting. Subsequently, a mixed-methods evaluation was performed. | **Quantitative and Qualitative:**  Questionnaire, semi-structured interview  After the 14-day usage period, patients were asked to fill out the questionnaires. | **Patients:**  8 participants (f: 0) | - Validity - Usability - Technology - Use & Adherence - Doctor-patient relationship - Individuality - Implementation - Costs | - |
