# Supplementary material for: Problems and Barriers Related to the Use of Digital Health Applications: Scoping Review
Source: J Med Internet Res. 2023 May 12;25:e43808. doi: 10.2196/43808 (PMC10221513; doi:10.2196/43808)
Supplement: Multimedia Appendix 2 [file jmir_v25i1e43808_app2.docx]

## Appendix 2: Problems mentioned in articles

|  | **Validity** | **Usability** | **Techno-logy** | **Use & Adher-ence** | **Data Security & Data Privacy** | **Doctor-patient relationship** | **Know-ledge & Skills** | **Indivi-duality** | **Implemen-tation** | **Costs** | **Total** |
| --- | --- | --- | --- | --- | --- | --- | --- | --- | --- | --- | --- |
| **Argent et al. (2018) [16]** | ● | ● | ● | ● | ● | ● | ● | ● | ● |  | 9 |
| **Barceló-Soler et al. (2019) [17]** |  |  |  | ● |  |  | ● | ● | ● |  | 4 |
| **Beer et al. (2020) [18]** | ● | ● | ● | ● | ● |  | ● |  |  | ● | 7 |
| **Bentley et al. (2020) [19]** |  | ● | ● | ● |  |  | ● | ● |  |  | 5 |
| **Browne et al. (2020) [20]** |  | ● | ● | ● |  |  |  |  |  |  | 3 |
| **Bucci et al. (2019) [21]** | ● |  |  | ● | ● | ● | ● | ● | ● | ● | 8 |
| **Chung et al. (2020) [22]** |  | ● | ● | ● |  |  | ● |  | ● |  | 5 |
| **Cuijpers et al. (2017) [23]** | ● |  |  |  |  | ● | ● |  | ● |  | 4 |
| **Huckvale et al. (2015) [24]** | ● |  | ● | ● |  |  | ● |  | ● |  | 5 |
| **Kowatsch et al. (2021) [25]** | ● | ● | ● | ● |  | ● |  | ● |  |  | 6 |
| **Luna-Perejon et al. (2019) [26]** | ● | ● | ● | ● |  |  |  | ● |  |  | 5 |
| **Minen et al. (2021) [27]** | ● |  | ● |  | ● | ● | ● |  |  | ● | 6 |
| **Mohr et al. (2021) [28]** | ● |  |  | ● | ● |  |  |  | ● | ● | 5 |
| **Okorodudu et al. (2015) [29]** | ● | ● | ● | ● | ● | ● | ● | ● | ● | ● | 10 |
| **Possemato et al. (2017) [30]** | ● | ● |  | ● |  | ● | ● |  | ● |  | 6 |
| **Pratap et al. (2018) [31]** |  |  |  | ● |  | ● |  |  | ● | ● | 4 |
| **Ravn Jakobsen et al. (2018) [32]** |  |  |  |  |  | ● |  |  |  |  | 1 |
| **Sobrinho et al. (2018) [33]** | ● | ● | ● |  | ● |  | ● | ● |  |  | 6 |
| **Son et al. (2020) [34]** |  |  |  |  | ● | ● | ● |  |  | ● | 4 |
| **Sun et al. (2017) [35]** | ● | ● |  | ● | ● |  | ● | ● | ● | ● | 8 |
|  | **Validity** | **Usability** | **Techno-logy** | **Use & Adher-ence** | **Data Security & Data Privacy** | **Doctor-patient relationship** | **Know-ledge & Skills** | **Indivi-duality** | **Implemen-tation** | **Costs** | **Total** |
| **Wirken et al. (2018) [36]** |  |  |  |  |  |  |  | ● |  |  | 1 |
| **Chen et al. (2016) [37]** |  | ● | ● | ● |  |  | ● | ● |  |  | 5 |
| **Hatcher et al. (2018) [38]** |  | ● | ● | ● |  | ● |  |  | ● |  | 5 |
| **Skar et al. (2017) [39]** | ● | ● | ● | ● |  |  | ● | ● | ● |  | 7 |
| **Kellett et al. (2020) [40]** | ● | ● | ● | ● | ● | ● |  | ● | ● |  | 8 |
| **Hardy et al. (2018) [41]** |  | ● |  |  | ● |  | ● |  | ● |  | 4 |
| **Thies et al. (2017) [42]** |  | ● | ● | ● | ● |  | ● |  | ● | ● | 7 |
| **Thirumalai et al. (2018) [43]** |  | ● | ● | ● |  |  |  | ● |  |  | 4 |
| **Woods et al. (2019) [44]** | ● | ● | ● | ● |  | ● |  | ● | ● | ● | 8 |
| **Total** | 16 | 19 | 18 | 22 | 12 | 13 | 18 | 15 | 17 | 10 | 160 |
