# Supplementary material for: Problems and Barriers Related to the Use of Digital Health Applications: Scoping Review
Source: J Med Internet Res. 2023 May 12;25:e43808. doi: 10.2196/43808 (PMC10221513; doi:10.2196/43808)
Supplement: Multimedia Appendix 3 [file jmir_v25i1e43808_app3.docx]

## Appendix 3: Studies assessed in the full-text screening and exclusion criteria

| **No.** | **Author(s)** | **Year** | **Title** | **Country** | **Study type** | **Inclusion / Exclusion** | **Reason for exclusion** |
| --- | --- | --- | --- | --- | --- | --- | --- |
| 1 | Akbar, Coiera & Magrabi [45] | 2020 | Safety concerns with consumer-facing mobile health applications and their consequences: a scoping review | Australia | Scoping Review | Exclusion | No relevant problems |
| 2 | Ana et al. [46] | 2020 | Mobile applications in oncology: A systematic review of health science database | Spain | Systematic Review | Exclusion | Not similar to DiGA |
| 3 | Argent et al. [16] | 2018 | Clinician perceptions of a prototype wearable exercise biofeedback system for orthopaedic rehabilitation: a qualitative exploration | Ireland | Semi-structured interviews | Inclusion | - |
| 4 | Armontrout et al. [47] | 2018 | Current Regulation of Mobile Mental Health Applications | USA | Review | Exclusion | Not similar to DiGA |
| 5 | Barceló-Soler et al. [17] | 2019 | Interventions of computerized psychotherapies for depression in Primary Care in Spain | Spain | Narrative review | Inclusion | - |
| 6 | Beer et al. [18] | 2020 | A Focus Group Evaluation of Breathe Easier: A Mindfulness-Based mHealth App for Survivors of Lung Cancer and Their Family Members | USA | Semi-structured focus groups | Inclusion | - |
| 7 | Bentley et al. [19] | 2020 | The Use of a Smartphone App and an Activity Tracker to Promote Physical Activity in the Management of Chronic Obstructive Pulmonary Disease: Randomized Controlled Feasibility Study | UK | Patients: Randomized controlled trial (RCT) and semi-structured interviews.  Questionnaire-based and physical activity-based outcome measures were taken at baseline, the end of pulmonary rehabilitation, and the end of maintenance.  Health care professionals: interviews and focus group discussion. | Inclusion | - |
| 8 | Browne et al. [20] | 2020 | Mobile Health Apps in Pediatric Obesity Treatment: Process Outcomes From a Feasibility Study of a Multicomponent Intervention | Ireland | RCT, questionnaires and discussions | Inclusion | - |
| 9 | Bucci et al. [21] | 2019 | "They Are Not Hard-to-Reach Clients. We Have Just Got Hard-to-Reach Services." Staff Views of Digital Health Tools in Specialist Mental Health Services | UK | Semi-structured focus groups | Inclusion | - |
| 10 | Castelnuovo et al. [48] | 2015 | Managing chronic pathologies with a stepped mHealth-based approach in clinical psychology and medicine | Italy | Review | Exclusion | Not similar to DiGA |
| 11 | Christopoulos et al. [49] | 2017 | Lessons learned from the implementation of seek, test, treat, retain interventions using mobile phones and text messaging to improve engagement in HIV care for vulnerable populations in the United States | USA | Review | Exclusion | Not similar to DiGA |
| 12 | Chung et al. [22] | 2020 | Mobile App Use for Insomnia Self-Management in Urban Community-Dwelling Older Korean Adults: Retrospective Intervention Study | Korea | Questionnaires | Inclusion | - |
| 13 | Claborn et al. [50] | 2018 | Improving adoption and acceptability of digital health interventions for HIV disease management: a qualitative study | USA | Focus Groups | Exclusion | Not similar to DiGA |
| 14 | Cuijpers et al. [23] | 2017 | Internet and mobile interventions for depression: Opportunities and challenges | Netherlands | Narrative review | Inclusion |  |
| 15 | Dithmer et al. [51] | 2016 | "The Heart Game": Using Gamification as Part of a Telerehabilitation Program for Heart Patients | Denmark | Interviews | Exclusion | No relevant problems |
| 16 | Dunne et al. [52] | 2020 | Maximizing Telerehabilitation for Patients With Visual Loss After Stroke: Interview and Focus Group Study With Stroke Survivors, Carers, and Occupational Therapists | UK | Interviews and Focus Groups | Exclusion | Not similar to DiGA |
| 17 | Eno et al. [53] | 2019 | Perspectives on implementing mobile health technology for living kidney donor follow-up: In-depth interviews with transplant providers | USA | Interviews | Exclusion | Not similar to DiGA |
| 18 | Fiorini, De Giacomo & L’Abate [54] | 2015 | Towards Resilient Telehealth Support for Clinical Psychiatry and Psychology: A Strategic Review | Italy | Review | Exclusion | Not similar to DiGA |
| 19 | Foong et al. [55] | 2020 | Facilitators and barriers of using digital technology for the management of diabetic foot ulcers: A qualitative systematic review | Singapore | Qualitative Systematic Review | Exclusion | Not similar to DiGA |
| 20 | Forman et al. [56] | 2019 | Randomized controlled trial of OnTrack, a just-in-time adaptive intervention designed to enhance weight loss | USA | Randomized Controlled Trial | Exclusion | No relevant problems |
| 21 | Furlong et al. [57] | 2018 | Mobile apps for treatment of speech disorders in children: An evidence-based analysis of quality and efficacy | Australia | (Play- / Apple-) Store review | Exclusion | No relevant problems |
| 22 | Glynn et al. [58] | 2015 | Patients' views and experiences of technology based self-management tools for the treatment of hypertension in the community: A qualitative study | Ireland | Focus Groups | Exclusion | Not similar to DiGA |
| 23 | Gorini et al. [59] | 2018 | A P5 approach to m-Health: Design suggestions for advanced mobile health technology | Italy | Design Study | Exclusion | Not disease related |
| 24 | Grau et al. [60] | 2016 | [Assessment method for mobile health applications in Spanish: The iSYScore index] | Spain | Not in English, German or French | Exclusion | Language |
| 25 | Griffin & Kehoe [61] | 2018 | A questionnaire study to explore the views of people with multiple sclerosis of using smartphone technology for health care purposes | UK | Questionnaire Study | Exclusion | Not similar to DiGA |
| 26 | Halbron, Joubert & Sonnet [62] | 2016 | French-speaking m-health and diabetes: An update | France | Qualitative Research Review | Exclusion | Not similar to DiGA |
| 27 | Huckvale et al. [24] | 2015 | Smartphone apps for calculating insulin dose: a systematic assessment | UK | App assessment | Inclusion | - |
| 28 | Jacobson, Summers & Wilhelm [63] | 2020 | Digital Biomarkers of Social Anxiety Severity: Digital Phenotyping Using Passive Smartphone Sensors | Lebanon | Quantitative Research | Exclusion | Not similar to DiGA |
| 29 | Jimenez-Molina, Gaete-Villegas & Fuentes [64] | 2018 | ProFUSO: Business process and ontology-based framework to develop ubiquitous computing support systems for chronic patients' management | Chile | Framework Development | Exclusion | Not similar to DiGA |
| 30 | Jindal et al. [65] | 2018 | Development of mWellcare: an mHealth intervention for integrated management of hypertension and diabetes in low-resource settings | India | App development | Exclusion | Not used by patient(s) |
| 31 | Jogova, Shaw & Jamieson [66] | 2019 | The Regulatory Challenge of Mobile Health: Lessons for Canada | Canada | Review | Exclusion | Not disease related |
| 32 | Jones, DeRuyter & Morris [67] | 2020 | The Digital Health Revolution and People with Disabilities: Perspective from the United States | USA | Review | Exclusion | Not disease related |
| 33 | Kincaid [68] | 2021 | Implications of Hispanics using mhealth applications to self-manage diabetes: A quantitative, cross-sectional analysis | USA | Cross-Sectional Analysis | Exclusion | Not similar to DiGA |
| 34 | Kowatsch et al. [25] | 2021 | Hybrid Ubiquitous Coaching With a Novel Combination of Mobile and Holographic Conversational Agents Targeting Adherence to Home Exercises: Four Design and Evaluation Studies | Switzerland | Observational study, semi-structured interviews and survey | Inclusion | - |
| 35 | Kruse et al. [69] | 2019 | Barriers to the Use of Mobile Health in Improving Health Outcomes in Developing Countries: Systematic Review | USA | Systematic Review | Exclusion | Not similar to DiGA |
| 36 | Lhotska et al. [70] | 2016 | Non-technical Issues in Design and Development of Personal Portable Devices | Czech Republic | Review | Exclusion | Not disease related |
| 37 | Luna-Perejon et al. [26] | 2019 | Evaluation of user satisfaction and usability of a mobile app for smoking cessation | Spain / Greece / Taiwan | Observational study, questionnaires, expert reports | Inclusion | - |
| 38 | MacKinnon & Brittain [71] | 2020 | Mobile Health Technologies in Cardiopulmonary Disease | USA | Review | Exclusion | Not similar to DiGA |
| 39 | Maloney, Abel, McLeod [72] | 2020 | Jamaican adolescents' receptiveness to digital mental health services: A cross-sectional survey from rural and urban communities | Jamaica | Descriptive Cross-Sectional Feasibility Study | Exclusion | No relevant problems |
| 40 | Messner [73] | 2019 | mHealth applications: Potentials, limitations, current quality and future directions | Germany | Book Chapter | Exclusion | Not disease related |
| 41 | Meyerowitz-Katz et al. [74] | 2020 | Rates of Attrition and Dropout in App-Based Interventions for Chronic Disease: Systematic Review and Meta-Analysis | Australia | Systematic Review | Exclusion | Not similar to DiGA |
| 42 | Minen et al. [27] | 2021 | The Functionality, Evidence, and Privacy Issues Around Smartphone Apps for the Top Neuropsychiatric Conditions | USA | App assessment | Inclusion | - |
| 43 | Miranda et al. [75] | 2017 | Addressing post-stroke care in rural areas with Peru as a case study. Placing emphasis on evidence-based pragmatism | Peru | Review | Exclusion | Not similar to DiGA |
| 44 | Mohr et al. [28] | 2021 | Banbury Forum Consensus Statement on the Path Forward for Digital Mental Health Treatment | USA | Review within a forum | Inclusion | - |
| 45 | Morera et al. [76] | 2016 | Security Recommendations for mHealth Apps: Elaboration of a Developer's Guide | Spain | Developer’s Guide | Exclusion | Not similar to DiGA |
| 46 | Neubeck et al. [77] | 2017 | Is there an app for that? Mobile phones and secondary prevention of cardiovascular disease | UK | Review | Exclusion | Not similar to DiGA |
| 47 | Ng et al. [78] | 2019 | User Engagement in Mental Health Apps: A Review of Measurement, Reporting, and Validity | USA | Review | Exclusion | No relevant problems |
| 48 | Nightingale et al. [79] | 2017 | Desirable Components for a Customized, Home-Based, Digital Care-Management App for Children and Young People With Long-Term, Chronic Conditions: A Qualitative Exploration | UK | Qualitative Exploration | Exclusion | Not similar to DiGA |
| 49 | Nouri et al. [80] | 2019 | Assessing Mobile Phone Digital Literacy and Engagement in User-Centered Design in a Diverse, Safety-Net Population: Mixed Methods Study | USA | Mixed Methods Study | Exclusion | Not similar to DiGA |
| 50 | Nymberg et al. [81] | 2019 | 'Having to learn this so late in our lives...' Swedish elderly patients' beliefs, experiences, attitudes and expectations of e-health in primary health care | Sweden | Focus Groups | Exclusion | Not similar to DiGA |
| 51 | Okorodudu, Bosworth & Corsino [29] | 2015 | Innovative interventions to promote behavioral change in overweight or obese individuals: A review of the literature | USA | Semi-structured review | Inclusion | - |
| 52 | Pedersen et al. [82] | 2019 | Predicting Dropouts From an Electronic Health Platform for Lifestyle Interventions: Analysis of Methods and Predictors | Denmark | Analysis of Methods and Predictors | Exclusion | Not similar to DiGA |
| 53 | Peprah et al. [83] | 2020 | Lessening barriers to healthcare in rural Ghana: providers and users' perspectives on the role of mHealth technology. A qualitative exploration | Ghana | Qualitative Exploration | Exclusion | Not disease related |
| 54 | Possemato et al. [30] | 2017 | Development and refinement of a clinician intervention to facilitate primary care patient use of the PTSD Coach app | USA | Feedback, questionnaire and interview | Inclusion | - |
| 55 | Pratap et al. [31] | 2018 | Using Mobile Apps to Assess and Treat Depression in Hispanic and Latino Populations: Fully Remote Randomized Clinical Trial | USA | Questionnaires and passive data collection | Inclusion | - |
| 56 | Ravn Jakobsen et al. [32] | 2018 | Development of an mHealth Application for Women Newly Diagnosed with Osteoporosis without Preceding Fractures: A Participatory Design Approach | Denmark | Field studies and semi-structured interviews | Inclusion | - |
| 57 | Roberts et al. [84] | 2019 | Breast, Prostate, and Colorectal Cancer Survivors' Experiences of Using Publicly Available Physical Activity Mobile Apps: Qualitative Study | UK | Qualitative study | Exclusion | Not similar to DiGA |
| 58 | Safdari, abadi & Nejad [85] | 2018 | Improve health of the elderly people with M-health and technology | Iran | Review | Exclusion | Not disease related |
| 59 | Sahin [86] | 2018 | Rules of engagement in mobile health: what does mobile health bring to research and theory? | Canada | Review | Exclusion | Not disease related |
| 60 | Saner & Van Velde [87] | 2016 | eHealth in cardiovascular medicine: A clinical update | Swiss / Netherlands | Clinical Update | Exclusion | Not similar to DiGA |
| 61 | Silva et al. [88] | 2015 | Mobile-health: A review of current state in 2015 | Portugal | Review | Exclusion | Not disease related |
| 62 | Smith & Magnani [89] | 2019 | New technologies, new disparities: The intersection of electronic health and digital health literacy | USA | Review | Exclusion | Not similar to DiGA |
| 63 | Sobrinho et al. [33] | 2018 | Design and evaluation of a mobile application to assist the self-monitoring of the chronic kidney disease in developing countries | Brazil | Interviews, questionnaire, observation | Inclusion | - |
| 64 | Son et al. [34] | 2020 | Patients' needs and perspectives for using mobile phone interventions to improve heart failure self-care: A qualitative study | Korea | Semi-structured interview | Inclusion | - |
| 65 | Sun et al. [35] | 2017 | Facilitators and barriers to using physical activity smartphone apps among Chinese patients with chronic diseases | China | Questionnaires | Inclusion | - |
| 66 | Terhorst et al. [90] | 2021 | Systematic evaluation of content and quality of English and German pain apps in European app stores | Germany | Systematic Evaluation | Exclusion | Not similar to DiGA |
| 67 | Tokgöz et al. [91] | 2021 | Digital health interventions in prevention, relapse, and therapy of mild and moderate depression: Scoping review | Germany | Scoping Review | Exclusion | No relevant problems |
| 68 | Williams et al. [92] | 2021 | Addressing Implementation Challenges to Digital Care Delivery for Adults With Multiple Chronic Conditions: Stakeholder Feedback in a Randomized Controlled Trial | USA | Randomized Controlled Trial | Exclusion | Not similar to DiGA |
| 69 | Wirken et al. [36] | 2018 | Development and feasibility of a guided and tailored internet-based cognitive-behavioural intervention for kidney donors and kidney donor candidates | Netherlands | Semi-structured focus groups, questionnaires, technical data (eg. The frequency of logins) | Inclusion | - |
| 70 | Yin et al. [93] | 2020 | Mobile Mental Health Apps in China: Systematic App Store Search | China | Systematic App Store Search | Exclusion | Not similar to DiGA |
| 71 | Zhang et al. [94] | 2017 | The role of perceived e-health literacy in users' continuance intention to use mobile healthcare applications: An exploratory empirical study in China | China | Exploratory Study | Exclusion | Not disease related |
| 72 | Zhao et al. [95] | 2019 | Mobile applications for pain management: an app analysis for clinical usage | USA | App Analysis | Exclusion | Not similar to DiGA |
